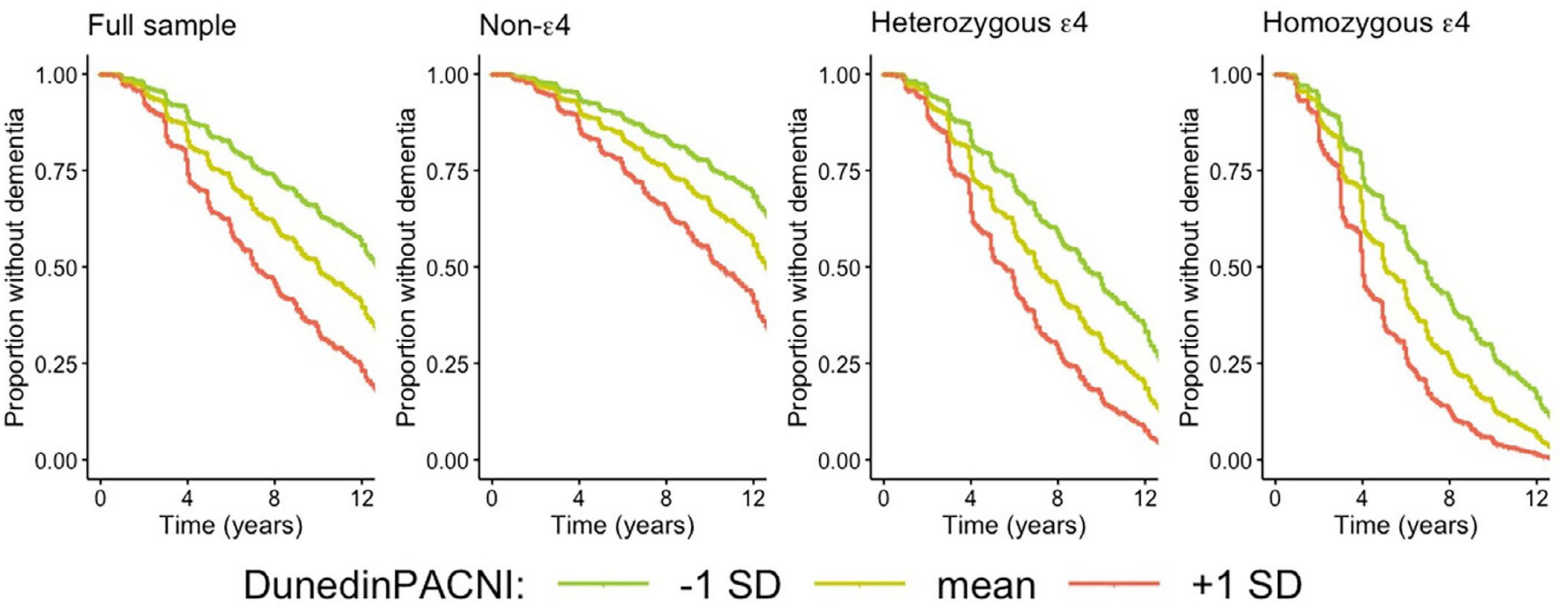# A brain MRI estimate of whole‐body aging in midlife predicts late life risk for Alzheimer’s disease

**DOI:** 10.1002/alz.084020

**Published:** 2025-01-09

**Authors:** Ethan T. Whitman, Maxwell L. Elliott, Annchen R. Knodt, Terrie E. Moffitt, Avshalom Caspi, Ahmad R. Hariri

**Affiliations:** ^1^ Duke University, Durham, NC USA; ^2^ Harvard University, Boston, MA USA

## Abstract

**Background:**

Aging is the strongest risk factor for Alzheimer’s disease (AD). Accordingly, identifying biomarkers of accelerated aging is a major focus of AD prevention research. Current MRI‐based “aging clocks” (i.e., brain age) derive from cross‐sectional associations with chronological age. However, chronological age differs fundamentally from biological aging. We introduce a novel brain MRI proxy for biological aging in midlife developed in the Dunedin Study, a population‐representative longitudinal birth cohort, and demonstrate its ability to differentiate and predict AD in the Alzheimer’s Disease Neuroimaging Initiative (ADNI).

**Method:**

The pace of whole‐body aging was determined by tracking physiological decline of 6 organ systems over 20 years in Dunedin Study members. An elastic‐net regression model was trained to estimate this pace of aging using a single T1‐weighted scan collected at age 45 from 860 Study members. We call this measure the Dunedin Pace of Aging Calculated from NeuroImaging or “DunedinPACNI.” We exported DunedinPACNI to ADNI and tested its association with current and future AD. Lastly, we compared DunedinPACNI to “brain age” using brainageR.

**Result:**

DunedinPACNI estimated whole‐body aging with a cross‐validated accuracy of r=0.43 in Dunedin Study members. Derived in 6,199 T1‐weighted ADNI scans, DunedinPACNI was on average 0.40 standard deviations higher in MCI patients and 1.13 standard deviations higher in AD patients compared to healthy participants (MCI 95% CI:[0.36‐0.45]; AD 95% CI:[1.08‐1.19]). DunedinPACNI outperformed brainageR in differentiating MCI and AD patients from controls (MCI: b=.25; 95% CI:[.14‐.35]; AD: b=.68; 95% CI:[.56‐.80]). In 1,326 ADNI participants followed up to 16 years, baseline DunedinPACNI was associated with a higher probability of AD conversion (HR=1.58, 95% CI:[1.36‐1.84]) as was brainageR (HR=1.61; 95% CI:[1.42‐1.84]). DunedinPACNI and brainageR together predicted conversion more accurately than either measure alone (combined HR=2.10, 95% CI:[1.76‐2.51).

**Conclusion:**

DunedinPACNI, a novel MRI proxy for longitudinal whole‐body aging during midlife, offers advantage over “brain age” in detecting neurodegenerative disease and predicting AD conversion in late life. DunedinPACNI can be applied to existing neuroimaging datasets to estimate whole‐body aging to better gauge early AD risk.